# Understanding the Failure Mechanism of Thermal Barrier Coatings Considering the Local Bulge at the Interface between YSZ Ceramic and Bond Layer

**DOI:** 10.3390/ma15010275

**Published:** 2021-12-30

**Authors:** Zhi-Yuan Wei, Hong-Neng Cai

**Affiliations:** State Key Laboratory for Mechanical Behavior of Materials, School of Materials Science and Engineering, Xi’an Jiaotong University, Xi’an 710049, China; zhiyuanwei@mail.xjtu.edu.cn

**Keywords:** thermal barrier coatings (TBCs), interface morphology, bulge, crack propagation, failure mechanism

## Abstract

The TC/BC interface morphology in APS TBC is one of the important factors leading to crack propagation and coating failure. Long cracks are found near the bulge on the TC/BC interface. In this study, the TBC model with the bulge on the interface is developed to explore the influence of the bulge on the coating failure. Dynamic TGO growth and crack propagation are considered in the model. The effects of the bulge on the stress state and crack propagation in the ceramic layer are examined. Moreover, the effects of the distribution and number of bulges are also investigated. The results show that the bulge on the interface results in the redistribution of local stress. The early cracking of the ceramic layer occurs near the top of the bulge. One bulge near the peak or valley of the interface leads to a coating life reduction of about 75% compared with that without a bulge. The increase in the number of bulges further decreases the coating life, which is independent of the bulge location. The results in this work indicate that a smooth TC/BC interface obtained by some possible surface treatments may be an optional scenario for improving coating life.

## 1. Introduction

Thermal barrier coatings (TBCs) are mainly coated on the high-temperature hot components for aero engines or gas turbines, such as combustion chambers, blades, and fuel nozzles. The thermal insulation of the ceramic material enables the substrate materials to be used at temperatures above their melting point [1,2,3]. The typical TBC system consists of two layers: ceramic top coat (TC) and bond coat (BC). The role of the BC layer is to reduce the thermal mismatch between ceramic and substrate and protect the substrate from oxidation. Atmospheric plasma spraying (APS) and electron beam physical vapor deposition (EB-PVD) are two popular methods to prepare coatings. The TBC obtained by EB-PVD has typical columnar structure feature [4,5,6]. The TBC deposited by APS is realized in a manner that the molten ceramic particles impact the substrate surface at high speed [7,8]. Before the APS coating is deposited, the substrate needs to be sandblasted to obtain a rough surface. Therefore, the TC/BC interface is often undulated, which can improve the bond strength between the coating and substrate. The compound 8 wt.% yttria partially stabilized zirconia (8YSZ) is often used for the TC layer. However, 8YSZ may undergo phase transition after long service at high temperature. Therefore, a double ceramic structure composed of new and traditional materials has been developed to improve the coating life [9,10,11].

The TBC system has long been in service at high temperature. Since the ceramic material is almost oxygen-transparent, the Al ions in the BC layer react with the O ions from the air at high temperature to form thermally grown oxide (TGO). The TGO thickness increases with the oxidation time [12,13]. The growth of TGO perpendicular to the TC/BC interface leads to its thickening. However, the growth parallel to the interface results in its constrained elongation. The high-temperature oxidation of the bond layer is responsible for the failure of the coating [14,15,16,17,18,19]. Since the TC/BC interface is rough in the APS TBC, the lateral growth of TGO will cause the interface amplitude change [20,21,22]. The stress near the TC/BC interface also accumulates as the TGO layer grows [23,24]. When the stress value exceeds the allowable stress of ceramic materials, crack propagation occurs in the ceramic layer. Note that corrosion failure, such as CMAS, is also an important mechanism of coating failure [25,26,27,28,29,30].

The interface morphology between TC and BC severely affects the life of APS TBC. In order to obtain the optimal interface morphology, it is necessary to understand the influences of interface morphology characteristics on the stress distribution in the coating. The finite element method can be used for parametric analysis of interface morphology [31,32,33,34,35]. Ranjbar-far et al. [36] used the cosine and semicircular curves to describe the TC/BC interface, respectively. They found that the interface morphology had little effect on the stress distribution. The interface roughness severely affected the stress state in the ceramic layer. Gupta et al. [37] established a TBC model based on the real interface morphology and analyzed the effect of TGO growth on the stress state at the interface. It is found that the normal tensile stress appears at the peak and the compressive stress at the valley when the TGO is thin. With the increase in TGO thickness, the stress state at the peak and valley is reversed. Zhu et al. [38] studied the effect of interface amplitude and wavelength ratio on the stress state by a model including real interface morphology. It is found that the stress amplitude extended with the increase in the ratio. In addition, several TBC models containing a three-dimensional interface were also developed to explore the stress characteristics in the coating [39,40,41]. The results indicated that the rough TC/BC interface led to huge stress in the local regions, especially when TGO growth was considered.

The huge stress at the interface means that crack initiation can occur. In fact, there are a large number of two-dimensional pores between and inside the lamellae for the APS TBC, which act as cracks. The coating failure is caused by the propagation and coalescence of these cracks. In order to clarify how the TC/BC interface affects the coating failure, the influences of interface characteristic on the crack driving force and propagation behavior must be explored. Hille et al. [42] explored the effects of imperfection at the interface on the coating failure and indicated that coating failure could be delayed by surface treatment. Ranjbar-far et al. [43] developed the TBC models with different interface morphologies to study the effects of the interface on crack evolution. Their results showed that the non-uniform interface led to longer crack than the uniform interface. Zhang et al. [44] investigated the interface roughness effects on surface crack propagation. They discovered that the strain energy at the peak and valley were different, and the fluctuation of the interface may cause the local arrest of surface cracks. Song et al. [45] examined the competitive mechanism of cracks at the interface and in the ceramic under different interface roughness, and found that a rougher interface may result in different failure modes and reduce the coating life. The above results indicated that the interfacial roughness seriously affected the crack propagation near the TC/BC interface.

Based on the studies of the interface morphology effects on the stress state or crack evolution, some interface optimization theories or methods have also been proposed. Yu et al. [46] established a mixed interface parameter containing both wavelength and amplitude to describe the interface influence on the stress state. Ranjbar-far et al. [47] considered that a uniform interface morphology was beneficial to the improvement of the coating spalling resistance. The results by Aktaa et al. [48] showed that when the amplitude of the cosine interface with 60 μm wavelength was 10.0 μm, the crack driving force was minimized. Weeks et al. [49] thought that the ratio of amplitude to wavelength can be optimized to delay the ceramic cracking and coating spallation. So they experimentally optimized the roughness of the TC/BC interface and found that the interface morphology with 15.0 μm ± 3.0 μm roughness, 66° ± 3° slope, and 120.0 μm ± 10.0 μm spacing could improve the TBC lifetime.

**Figure 1:**
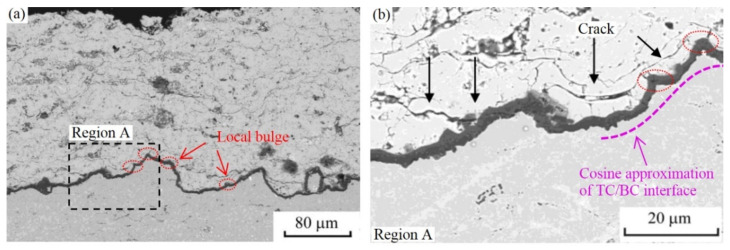
The cross-sectional morphology of APS TBC [50]: (**a**) local bulges at the TC/BC interface and (**b**) a magnification perspective of image in the region A.

The TC/BC interface in the APS TBC is not completely smooth. Some local bulges appear on the interface, as shown in Figure 1 [50]. The rough interfaces can lead to changes of local stress. Huge tensile and shear stresses may appear near the bulge, which will induce crack initiation and subsequent propagation. Therefore, early ceramic layer cracking can be found near the bulge of the interface [51]. In a word, these local bulges may contribute to the coating failure. It can be clearly seen from Figure 1b that some ceramic cracks appear near the local bulges, which indicate that the local bulge may cause the early propagation of cracks. In order to develop advanced TBC with long life, it is urgent to understand the effects of the local bulge on the stress state and ceramic cracking.

In this work, a TBC model with the bulge on the TC/BC interface was developed to study its failure mechanism under the cyclic thermal loading. The dynamic growth of TGO and the dynamic propagation of cracks are considered in the model. The effects of the bulge on the stress characteristic in the ceramic layer are first examined. Then, the dynamic propagation behavior of cracks in the coating containing the interface bulge was explored. In addition, the influences of the location and number of the bulge on the crack evolution and coating failure were also investigated.

## 2. Numerical Model Development

### 2.1. Geometry and Meshing

**Figure 2:**
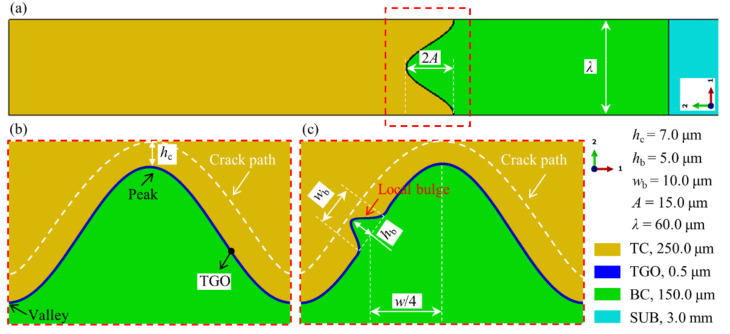
Geometric model of APS TBC: (**a**) overall model, (**b**) the TC/BC interface without bulge, and (**c**) the TC/BC interface with one bulge.

Here, the APS TBC is sprayed on nickel-based superalloy substrate (SUB) of 3.0 mm thickness. Figure 2 demonstrates the geometric model of the TBC system. The thicknesses of TC and BC are about 250.0 μm and 150.0 μm, respectively. The overall model is shown in Figure 2a. The interface morphology between TC and BC is often approximated by a cosine curve [23,36,45,52]. In this work, a cosine wave is investigated. Its wavelength and amplitude are 60.0 μm and 15.0 μm, respectively. The ceramic layer prepared by APS is stacked by lamellae. The inter-lamella crack near the TC/BC interface tends to be parallel to the interface. The crack propagation occurs under thermal shock condition [53,54]. Therefore, the cracking path in the ceramic layer in this study is considered to be parallel to the TC/BC interface. Figure 2b shows the cracking path near the TC/BC interface without bulge. Its normal distance to the interface is 7.0 μm. Figure 2c exhibits a geometrical model with one bulge in the middle of the TC/BC interface. The highest position of the bulge is perpendicular to the interface. Its height and bottom width are 5.0 μm and 10.0 μm, respectively. In this study, the bulge on the TC/BC interface has three locations: near-valley, middle, and near-peak. The distance between the bottom center points of these positions is 10.0 μm parallel to the interface. In order to prevent the early formation of mixed oxides during the thermal test, a uniform and dense *α*-Al_2_O_3_ layer is obtained after the coating deposition [12,55]. The initial TGO thickness in Figure 2 is 0.5 μm.

**Figure 3:**
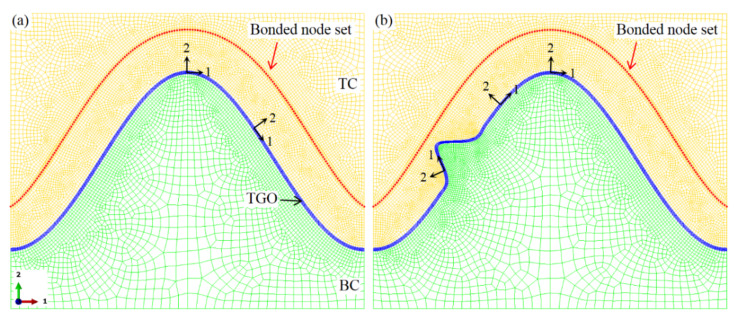
Finite element mesh near the TC/BC interface: (**a**) the mesh near the interface without bulge and (**b**) the mesh near the interface with one bulge.

The whole model is divided into quadrilateral grids. Since the ceramic cracking near the interface is considered, the dense meshes are used near the interface and cracking path, as shown in Figure 3. The characteristic element size is 0.45 μm. The generalized plane strain element with reduced integral (CPEG4R) is used to analyze the stress field and crack evolution in the coating. There is no element distortion caused by hourglass in all calculations.

The ceramic cracking simulation near the TC/BC interface is realized by the surface-based cohesive interaction in ABAQU software (6.14 version, Dassault systemes, Providence, RI, USA) [56]. This method does not require the existence of initial cracks. When the stress of the element exceeds the allowable stress of the material, the damage occurs. If the cumulative strain energy exceeds the fracture toughness, the node pairs appearing on the cracking path in Figure 3 are released to form cracks. This method has been used to investigate the interface damage in the TBC [57]. Detailed information about this method can be found [58]. In this study, the fracture stress of the ceramic layer is 100 MPa [43,59,60]. Its critical fracture energy is 10 J/m^2^ [38,61]. The maximum nominal stress criterion is used to describe the crack initiation. The power law is adopted to evaluate the mixed-mode behavior of crack. It is assumed that the components of fracture stress and fracture energy in all directions are equal [38,62]. The exponent of the power-law is 1.0 [45,54].

### 2.2. Material Property

8YSZ is used to prepare the TC layer; NiCoCrAlTaY is used to deposit the BC layer. All layers are considered to be isotropic. Because the TC layer is a brittle material, it is treated as an elastic material. The BC layer exhibits elastic-plastic behavior. When the system temperature is higher than 750 °C, its yield strength is 200 MPa. If the temperature is lower than 300 °C, the yield strength is 1000 MPa. If the temperature increases from 300 °C to 750 °C, the yield strength changes linearly [23,24]. The TGO layer can deform at high temperature, which can prevent its growth stress from being higher than the experimental value. However, it still exhibits elastic behavior at low temperatures [63]. When the temperature is below 900 °C, the yield strength of TGO is 10 GPa. If the system stays at a high temperature, its yield strength is 500 MPa. When the system temperature increases to high temperature from 900 °C, the yield strength changes linearly. Because the plastic deformation of the substrate has little effect on the stress state in the ceramic layer, the substrate is seen as an elastic material. Thermalmechanical parameters for all layers are shown in Table 1 [36,64]. Here, the creep behaviors of TGO, BC, and SUB at high temperature are also considered. Their creep parameters are demonstrated in Table 2 [36,47].

The thickness of TGO shows a parabolic growth law with oxidation time at high temperature [12]. The new oxides at the TC/BC interface lead to the thickening of TGO perpendicular to the TC/BC interface. However, the oxides formed between TGO grains make TGO exhibit lateral growth parallel to the interface. The growth of TGO is constrained by adjacent layers, which leads to large stress near the interface. In this study, TGO growth is realized by the swelling option in ABAQUS (Dassault systemes, Providence, RI, USA). Since TGO thickening follows a parabolic law, its dynamic growth must be implemented in the CREEP subroutine [56]. More information about TGO growth simulation can be found in the previous studies [65]. Lateral growth of TGO is an important factor affecting stress state and crack evolution [48]. Here, the ratio of lateral to thickening growth rate is set to 0.05 [52,62,66].

### 2.3. Boundary Condition

The TBC geometry in this study is a representative volume element of the whole sample. Therefore, the x-direction freedom of the left boundary in the model shown in Figure 2b is constrained. The right boundary is subjected to a periodic boundary condition by exerting a multipoint constraint. Such constraint can make the nodes on the right boundary have the same displacement in the x-direction. Meanwhile, they can move freely in the y-direction. In order to prevent the occurrence of rigid-body displacement during the calculation, the y-direction freedom of the substrate bottom is constrained. The ceramic surface is free.

### 2.4. Thermal Loading History

**Figure 4:**
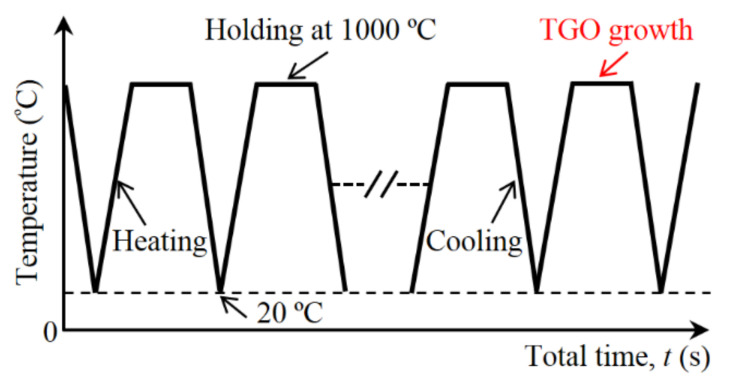
The cyclic thermal loading for all calculations (the number of thermal cycles is expressed by *N*).

The TBC system is often considered to be stress free at high temperature, which has been adopted in many studies [23,24,63,65]. In this study, the whole model is first cooled from 1000 °C to room temperature. Then it undergoes multiple thermal cycles. Finally, the model is reheated to a high temperature. The cyclic thermal loading for calculation is shown in Figure 4. A thermal cycle consists of three stages: heating, dwelling of 7200 s, and cooling. The TGO growth occurs in the dwelling stage. The effects of heating and cooling rate on the stress state are not considered here. Twenty cycles are investigated. The number of thermal cycles is expressed by *N*. A zero value for *N* represents the initial cooling process.

## 3. Results and Discussion

### 3.1. Stress Characteristics in the Ceramic Layer near the TC/BC Interface

Failure of APS TBC is the result of crack propagation and coalescence [38,53,54]. Both the normal stress *σ*_22_ and shear stress *σ*_12_ contribute to the propagation of cracks parallel to the interface in the coating [38,65]. Therefore, the research on the stress characteristics in this work mainly focus on these two types of stress.

**Figure 5:**
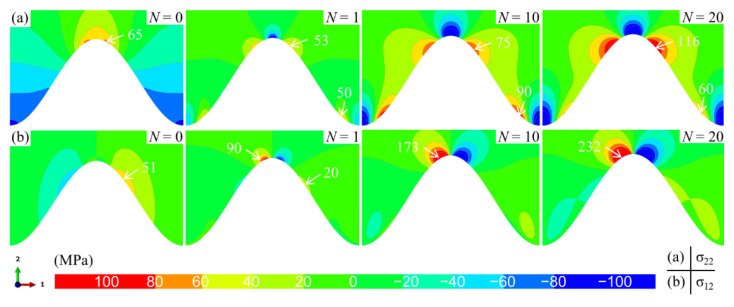
The stress distribution characteristic near the TC/BC interface without a bulge (at room temperature): (**a**) the normal stress *σ*_22_ and (**b**) the shear stress *σ*_12_.

Under the current condition, the stress distributions in the ceramic layer near the standard cosine interface without bulge are first studied, which is used to compare the results in the model with the bulge. Figure 5 shows the evolution of normal and shear stress distribution near the bulge-free interface with the thermal cycle. For the normal stress (see Figure 5a), when the model is cooled from the initial temperature to room temperature (*N* = 0), the tensile stress is located at the peak, and the valley is compressed, which is attributed to the larger shrinkage strain of the substrate compared with the ceramic layer. After one cycle (*N* = 1), the tensile stress shifts to both sides of the peak. As the thermal cycle progresses, the stress state near the interface basically no longer changes. However, the magnitude of tensile stress increases continuously, which is mainly attributed to the TGO growth at high temperature [23,45,62]. For the shear stress (see Figure 5b), it is always on both sides of the peak. Its magnitude increases with the thermal cycles.

**Figure 6:**
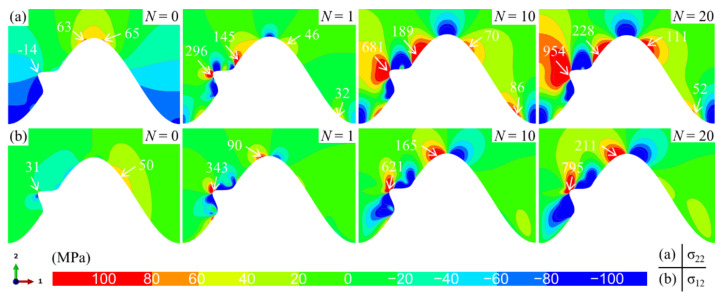
Distribution of the stress near the TC/BC interface with one bulge (at room temperature): (**a**) the normal stress *σ*_22_ and (**b**) the shear stress *σ*_12_.

When a bulge appears in the middle of the TC/BC interface, the stress state near the interface is changed, which is shown in Figure 6. The stress distribution on the left side of the model is significantly different from that in Figure 5. However, the stress distribution and amplitude on the right side of the model are basically consistent with the results in Figure 5. These indicate that the bulge only affects the local stress state in the ceramic layer but does not change the far-field stress characteristics. For the normal stress, there is huge tensile stress at the top of the bulge (see Figure 6a). With the progress of the thermal cycle, the tensile area and magnitude increase continuously. The shear stress also exhibits a similar trend (see Figure 6b).

**Figure 7:**
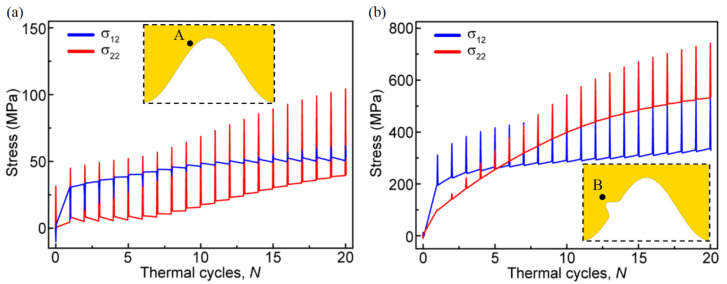
Variation of the normal and shear stress near the interface with the thermal cycles: (**a**) at point A near the interface without bulge and (**b**) at point B near the interface with one bulge.

In order to clarify the dynamic change process of stress in the ceramic layer, the variation of normal and shear stress near the interface with the number of thermal cycles are demonstrated in Figure 7. If no bulge occurs, the maximum tensile and shear stress in the ceramic layer appears near the peak. Therefore, the stress at point A is extracted (see Figure 7a). When there is a bulge on the interface, the maximum stress appears near the top of the bulge. Thus, the stress at point B is extracted (see Figure 7b). No matter whether a bulge occurs on the interface, the normal stress and shear stress in the ceramic layer near the interface shows a ratcheting increase with the thermal cycle. This is mainly due to the continuous accumulation of lateral growth strain in the TGO. In the model without bulge on the interface, the tensile and shear stress amplitudes in the ceramic layer are lower than those in the model with one bulge. In addition, it can be found that when there is a bulge on the interface, the stress in the ceramic layer has a higher increase rate. These mean that the emergence of a bulge on the interface will lead to the rapid increase in local stress to the cracking stress in a short time, which may induce the early cracking in the ceramic layer.

**Figure 8:**
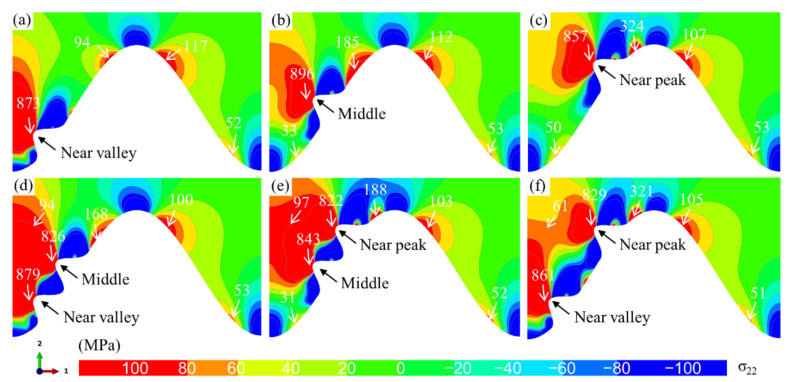
Effects of the location and number of bulges on the *σ*_22_ normal stress distribution (*N* = 20, at room temperature): (**a**–**c**) one bulge at the interface and (**d**–**f**) two bulges at the interface.

The distribution of bulges on the interface in APS TBC is not regular. Moreover, the number of bulges is not fixed. Therefore, it is necessary to study the influences of the location and number of bulges on the stress state in the ceramic layer. In this study, the bulge may appear at three locations on the interface: near-valley, middle, and near-peak. The normal stress distribution characteristics under different bulge configurations are demonstrated in Figure 8. It can be seen that the stress distribution in the right half of all models is not affected by the bulge. They show the same stress distribution and amplitude as the model with a standard cosine interface. When only one bulge appears (see Figure 8a–c), it can be found that there is also a tensile stress region between the bulge and interface peak. Moreover, when the bulge is at the near-peak, the tensile stress amplitude in this region is the largest. No matter where the bulge is, the maximum tensile stress in the ceramic layer always exists near the top of the bulge. When two bulges appear, the superposition of the stress field leads to a larger tensile stress area and magnitude, which is independent of the locations of the two bulges. These indicate that compared with the location, the number of bulges may more seriously affect the stress in the coating.

### 3.2. Crack Evolution in the Ceramic Layer near the TC/BC Interface

The change of stress distribution and magnitude in the ceramic layer can only indicate the ability of crack initiation or propagation. However, they cannot explain the influence of crack propagation on stress redistribution. Therefore, it is difficult to reveal coating failure directly by the stress state in the ceramic layer. In order to clarify the effect of the bulge on coating failure, the evolutions of the ceramic crack in the model with or without bulge on the interface are explored. It is noted that only when the damage initiation variable (CSMAXSCRT) reaches 1.0, the damage degradation (CSDMG) of the ceramic layer begins to increase from 0.0. If the CSDMG reaches 1.0, the node pairs on the crack surface begin to be released.

**Figure 9:**
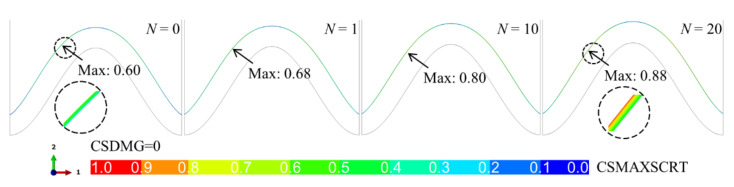
Evolution of damage initiation variable (CSMAXSCRT) in the ceramic layer near the interface without a bulge (at room temperature).

When there is no bulge on the interface, the stress in the ceramic layer does not increase much with the thermal cycle. After 20 cycles (*N* = 20), the stress magnitude is much lower than that in the model with a bulge (see Figure 7). This indicates that the possibility of ceramic cracking in the model without a bulge is much lower than that with a bulge. When there is no bulge on the interface, the low stress may not cause ceramic damage degradation, namely, CSDMG = 0. Figure 9 shows the variation of CSMAXSCRT along the cracking path in the model without a bulge. It is found that the maximum damage position appears on the left side of the peak, which is the same as the position of the maximum stress. The value of CSMAXSCRT increases continuously with the thermal cycle. After 20 cycles (*N* = 20), the maximum value is 0.88, indicating that the ceramic cracking does not occur in the model without a bulge.

**Figure 10:**
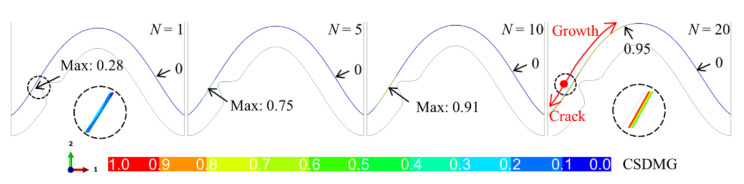
Evolution of damage degradation variable (CSDMG) in the ceramic layer near the interface including one bulge (at room temperature).

When there is a bulge in the middle of the interface, the huge tensile and shear stress near the bulge leads to damage degradation in the ceramic layer, as shown in Figure 10. It can be seen that when the system undergoes one thermal cycle (*N* = 1), the value of CSDMG near the top of the bulge is 0.28, which indicates that the damage degradation in the ceramic layer has occurred. The value of CSDMG increases with thermal cycling. When the cycle number reaches 10 (*N* = 10), the CSDMG has reached 0.91, which means that the ceramic cracking is about to occur. When the system undergoes more thermal cycles, the crack appears in the coating. It expands to the valley and peak. In a limited thermal cycle, the crack quickly propagates to the valley. Subsequently, the crack continues to expand to the peak. When the cycle number is equal to 20 (*N* = 20), the crack propagates to the near-peak. As the thermal cycle progresses, the CSDMG value in the right half of the model is always zero, indicating that the ceramic cracking in the left side does not affect the right side.

**Figure 11:**
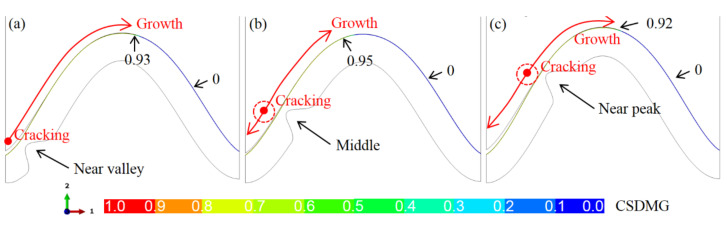
The CSDMG distributions in the ceramic layer under different locations of one bulge (*N* = 20, at room temperature): (**a**) near the valley, (**b**) middle, and (**c**) near the peak.

Because the ceramic crack first appears near the top of the bulge, the location of the bulge may affect the initiation and total length of the crack. Figure 11 shows the crack propagation in the ceramic layer under different bulge locations (*N* = 20). It can be seen that no matter where the bulge is, the ceramic cracking first occurs near the top of the bulge. When the bulge is located in the middle of the interface, the crack propagation does not cross the peak. Moreover, the crack opening at the valley on the left side of the model is also significantly smaller than that in the model with a bulge near the valley or peak.

**Figure 12:**
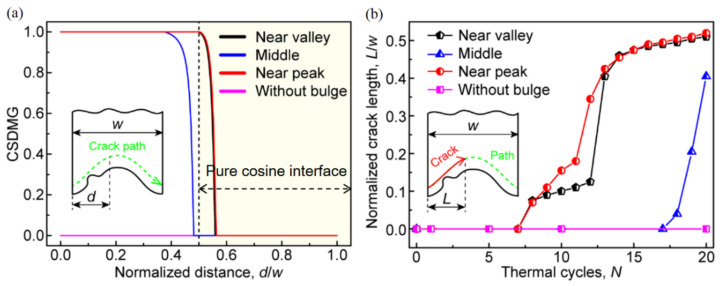
Effects of the location of one bulge on the ceramic cracking: (**a**) the CSDMG along the cracking path in the ceramic layer (*N* = 20, at room temperature) and (**b**) variation of the total crack length with the thermal cycles.

Besides the total length of the crack, the bulge location may also affect the cracking moment. Figure 12 shows the CSDMG distribution on the crack path and the change of the total crack length with the thermal cycles. It can be seen from Figure 12a that if there is no bulge on the interface, the CSDMG on the whole cracking path is 0.0. When the bulge is located near the valley or peak, the cracking area (CSDMG = 1.0) is significantly larger than that with a bulge in the middle of the interface. In addition, the damage degradation region (CSDMG > 0.0) on the cracking path is also obviously larger. It can be found from Figure 12b that the existence of bulge leads to the rapid increase in crack length, which is independent of the location of the bulge. When crack propagates to the near-peak region, the effect of the bulge is obviously weakened, so the total length of the crack increases at a lower rate with the thermal cycling.

**Figure 13:**
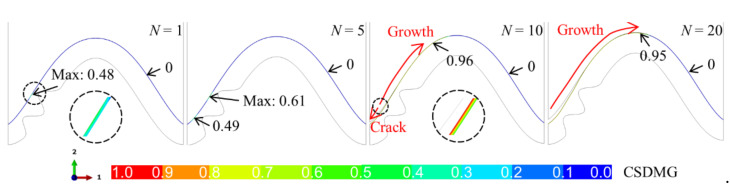
Evolution of the CSDMG in the ceramic layer near the interface including two bulges (at room temperature).

Based on the above stress results, it can be seen that the superposition of the bulge-induced stress field will further increase the local tensile stress region and magnitude. This indicates that the number of bulges may affect the crack behavior in the ceramic layer. Figure 13 shows the evolution of cracks in the ceramic layer with the thermal cycles when bulge occurs at the near-valley and in the middle of the interface. It can be found that multiple damage regions appear at the same time (*N* = 5). When the number of thermal cycles reaches 10 (*N* = 10), the ceramic crack has initiated from the top of the bulge at the near-valley and expanded to the near-peak region. Note that when only one bulge is located in the middle of the interface, the ceramic cracking does not occur when the cycle number is equal to 10 (see Figure 10). In addition, it can also be seen from Figure 13 that the crack in the ceramic layer has extended to the peak when the thermal cycle reaches 20. Obviously, the crack length is also higher than that when there is only a bulge in the middle of the interface. This means that the increase in the bulge number may lead to the earlier cracking and coating spalling.

**Figure 14:**
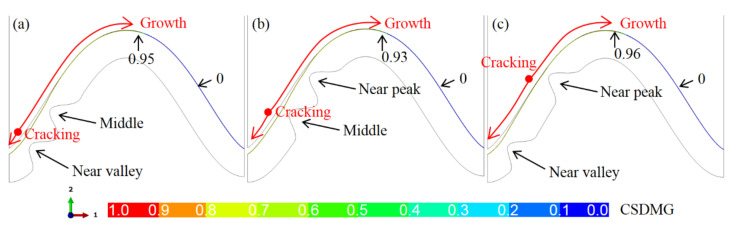
The CSDMG distributions in the ceramic layer under different locations of two bulges (*N* = 20, at room temperature): (**a**) near valley and middle, (**b**) middle and near peak, and (**c**) near valley and peak.

Figure 14 demonstrates the influence of the distribution of two bulges on the crack propagation in the ceramic layer. It is seen that no matter how the bulge is distributed, the cracks in the ceramic layer still initiate from near the top of the bulge. Under different bulge distributions, the cracks in the ceramic layer pass through the peak. They have a similar distribution. At the same time, it is also found that in the right region of the model, CSDMG is still 0.0, which is the same as that in the models with one or no bulge.

**Figure 15:**
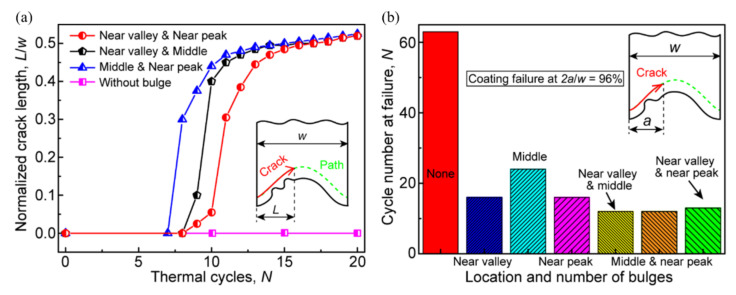
Effects of the location and number of bulges on the coating failure: (**a**) variation of the total crack length with the thermal cycles under different locations of two bulges and (**b**) the cycle number at the TBC failure under different bulge configurations.

In order to quantitatively reveal the effect of the bulge on the coating failure, Figure 15 shows the effects of the location and number of the bulge on the crack evolution. It can be seen from Figure 15a that the early cracking of the ceramic layer occurs after several cycles, which is independent of the distribution location of two bulges. Before the crack propagation reaches the peak, the crack length shows a sharp increase, which is different from the effect of a single bulge on crack propagation (see Figure 12b). Moreover, the crack propagation rate is also higher than that in the case of only a single bulge. It can be found from Figure 15b that when there is no bulge on the interface, the coating failure occurs after 63 cycles (*N* = 63). However, when a bulge appears at the near-peak or near-valley location, the coating failure occurs after 16 cycles (*N* = 16). If two bulges appear on the interface, the number of thermal cycles at failure will be further reduced, which is independent of the bulge distribution.

## 4. Conclusions

In this work, a TBC model with a bulge on the TC/BC interface is developed to explore the failure mechanism of the coating. A standard TBC model without bulge is also established for comparative analysis. The dynamic growth of TGO is included in the model through subroutine development. The initiation and propagation of cracks in ceramic layers are realized by the surface-based cohesive method. The effects of the bulge on the stress characteristic and crack evolution under cyclic thermal loading are implemented. The influences of bulge location and number on the coating failure are also explored. The main conclusions are as follows:

(1) The bulge results in the redistribution of local stress in the ceramic layer. Huge tensile and shear stress exists near the bulge.
(2) The superposition of stress regions induced by multiple bulges causes the further increase in tensile stress region and magnitude.
(3) The early ceramic cracking occurs near the top of the bulge. The bulge leads to a sharp increase in the crack length.
(4) One bulge near the peak or valley of the interface results in a 75% reduction in the coating life compared with the bulge-free model.
(5) The increase in the number of bulges will further decrease the coating life, which is independent of the bulge location.

## Figures and Tables

**Table 1:** Temperature-dependent thermal expansion coefficient *α*, Young’s modulus *E* and Poisson ratio *ν* data used for TC, TGO, BC, and SUB [36,64].

*T* (°C)	*E* (GPa)	*Ν*	*α* × 10^−6^ (°C^−1^)	*E* (GPa)	*Ν*	*α* × 10^−6^ (°C^−1^)
TC				TGO		
25	17.5	0.20	9.68	378	0.27	5.1
200	–	0.20	–	371	0.27	–
400	–	0.20	9.70	361	0.27	–
800	–	0.20	–	336	0.27	–
1000	12.4	0.20	10.34	311	0.27	9.8
BC				SUB		
25	218	0.30	10.3	210	0.30	–
200	209	0.30	11.3	200	0.30	12.6
400	199	0.30	12.5	187	0.30	13.6
800	162	0.30	14.3	156	0.30	15.4
1000	118	0.30	16.0	138	0.30	16.3

**Table 2:** Creep data for the TGO, BC, and SUB layer [36,47].

	*B* (s^−1^·MP^−n^)	*N*	*T* (°C)
TGO	7.3 × 10^−10^	1	1000
BC	6.54 × 10^−19^	4.57	≤600
	2.2 × 10^−12^	2.99	700
	1.8 × 10^−7^	1.55	800
	2.15 × 10^−8^	2.45	≥850
SUB	4.85 × 10^−36^	1	10
	2.25 × 10^−9^	3	1200

## Data Availability

Not applicable.
